# Macromolecular Engineering by Applying Concurrent Reactions with ATRP

**DOI:** 10.3390/polym12081706

**Published:** 2020-07-29

**Authors:** Yu Wang, Mary Nguyen, Amanda J. Gildersleeve

**Affiliations:** 1 Department of Chemistry, University of Louisiana at Lafayette, Lafayette, LA 70504, USA; amanda.gildersleeve1@louisiana.edu; 2 Institute for Materials Research and Innovation, University of Louisiana at Lafayette, Lafayette, LA 70504, USA; 3 Department of Chemical Engineering, University of Louisiana at Lafayette, Lafayette, LA 70504, USA; mary.nguyen1@louisiana.edu

**Keywords:** macromolecular engineering, ATRP, click chemistry, CuAAC, RAFT, ROMP, ROP, concurrent polymerization

## Abstract

Modern polymeric material design often involves precise tailoring of molecular/supramolecular structures which is also called macromolecular engineering. The available tools for molecular structure tailoring are controlled/living polymerization methods, click chemistry, supramolecular polymerization, self-assembly, among others. When polymeric materials with complex molecular architectures are targeted, it usually takes several steps of reactions to obtain the aimed product. Concurrent polymerization methods, i.e., two or more reaction mechanisms, steps, or procedures take place simultaneously instead of sequentially, can significantly reduce the complexity of the reaction procedure or provide special molecular architectures that would be otherwise very difficult to synthesize. Atom transfer radical polymerization, ATRP, has been widely applied in concurrent polymerization reactions and resulted in improved efficiency in macromolecular engineering. This perspective summarizes reported studies employing concurrent polymerization methods with ATRP as one of the reaction components and highlights future research directions in this area.

## 1. Introduction

Modern polymeric material design often involves precise macromolecular synthesis to achieve targeted macroscopic material properties. This concept is called macromolecular engineering which includes the rational design of the macromolecular structure, precise synthesis, assembly, and processing to supramolecular objects or nanodevices, detailed characterization, and theoretical modeling to aid and to optimize the macromolecular design procedure [1]. Precise tailoring of the molecular/supramolecular structure that leads to desirable molecular size, uniformity, topology, composition, and functionality is the core of macromolecular engineering [2]. The available tools for tailoring of the molecular architectures are controlled/living polymerization methods [3], click chemistry [4], supramolecular polymerization [5,6], self-assembly [7,8], among others.

Among these synthetic methods, atom transfer radical polymerization (ATRP) is a particularly popular one [9,10,11,12]. Same as other reversible deactivation radical polymerization (RDRP) methods [13], ATRP is tolerant to many functional groups and could be performed in an aqueous medium. The large variety of available monomers and the relatively easy handling make ATRP a very attractive method in functional polymeric material design [14,15,16]. Sometimes, the targeted molecular structure could not be synthesized by a single polymerization method, thus combining two or more synthetic methods is also a commonly adopted strategy in macromolecular engineering. ATRP can control the end and the side functional groups of polymer chains and is easy to switch to or to combine with other reaction mechanisms to obtain the targeted molecular architectures and functionalities [17,18,19,20].

**Figure 1:**
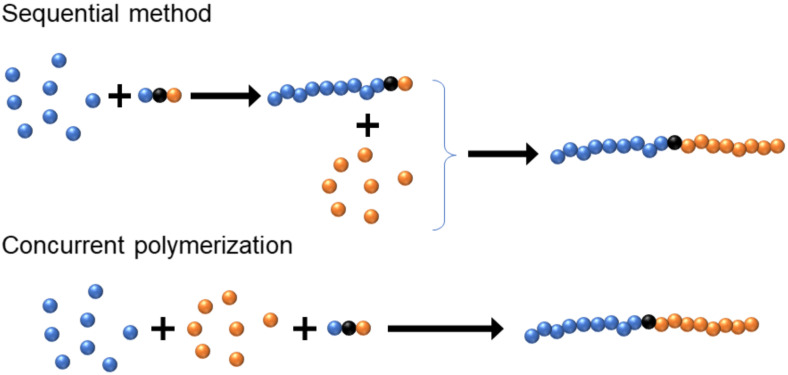
Procedures to synthesize a diblock copolymer.

However, increasing complexity of the targeted molecular architecture requires more and more complicated synthetic procedures to achieve the goal. As a result, concurrent polymerization, i.e., two or more reaction mechanisms take place simultaneously, is very attractive because it can significantly reduce the complexity of the synthesis. For example, the typical way to synthesize a diblock copolymer is (1) synthesis of a homopolymer by polymerizing one monomer; (2) purification of the homopolymer; (3) polymerizing the second monomer with the homopolymer as the macroinitiator; (4) purification of the diblock copolymer. The procedure can be simplified, if the two monomers are polymerized by different mechanisms, and the two orthogonal polymerization mechanisms do not interfere with each other, as (1) performing polymerization of both monomers simultaneously with a dual functional initiator; (2) purification of the product, Figure 1. In addition to simplifying the synthetic procedure, concurrent polymerization can also be used to obtain high-quality products or to synthesize special macromolecular architectures which would be otherwise very difficult to achieve. Successful concurrent polymerization methods provide very efficient and powerful tools for macromolecular engineering. Many concurrent reactions have been applied together with ATRP in advanced soft material design. This perspective summarizes the different types of concurrent reactions used together with ATRP illustrating the advantages of the concurrent polymerization methods. The future direction of this research area is highlighted at the end.

## 2. Concurrent Reactions Used Together with ATRP

ATRP employs a transition metal complex as the catalyst and an alkyl halide as the initiator (R-X). Various transition metal complexes, namely those of Cu, Ru, Fe, Ni, Os, etc. [21], have been employed as catalysts for ATRP. In an ATRP process, the dormant species R-X is activated by the transition metal complex to generate radicals via one electron transfer. Simultaneously the transition metal is oxidized to higher oxidation state. This reversible process rapidly establishes an equilibrium that is predominately shifted to the R-X side with very low radical concentrations. The number of polymer chains is determined by the number of R-X initiators. Each growing chain has the same probability to propagate with monomers to form living/dormant polymer chains (R-Pn-X). As a result, polymers with similar molecular weight and narrow molecular weight distribution can be prepared. In an ATRP system, the radical concentration, as well as the polymerization rate, depends on the activity of the catalyst, i.e., the ATRP equilibrium constant, and the molar ratio between the activator and the deactivator, i.e., [Cu(I)]/[Cu(II)] in Cu catalyzed ATRP. Thus, in principle the total amount of the catalyst should not affect the reaction rate. However, due to the radical termination, some of the activators would be converted permanently to deactivators [22]. To maintain the polymerization rate, relatively large initial amount of the activator, e.g., one equivalent to the alkyl halide initiator, is necessary. Alternatively, the activator can be regenerated by reduction of the deactivator. The continuous activator regeneration made it possible to use parts per million (ppm) catalyst loadings in ATRP reactions. These procedures include activator regeneration by electron transfer (ARGET) ATRP with additional reducing agents [11,23,24], initiators for continuous activator regeneration (ICAR) ATRP with conventional radical initiators [25,26], and supplemental activator and reducing agent (SARA) ATRP with zerovalent metals [27,28,29]. Non-chemical methods have also been developed including electrochemically mediated polymerization (*e*ATRP) [30,31], photochemically mediated polymerization (*photo*ATRP) [32], and mechanochemically mediated procedures (*mechano*ATRP) [33,34].

The concurrent reactions discussed here include polymerization or other coupling reactions taking place independently or interactively with ATRP, in situ generation of reagents for the polymerization, and concurrent functional group transition during the polymerization. The most commonly used polymerization mechanisms that can take place concurrently with ATRP are ring-opening polymerization (ROP) [35], ring-opening metathesis polymerization (ROMP) [36], reversible addition-fragmentation chain-transfer polymerization (RAFT) [37,38,39,40], step-growth polymerization by atom transfer radical addition (ATRA) [41]. Copper-catalyzed azide-alkyne cycloaddition (CuAAC) [42] click reaction can also be used concurrently with ATRP as a method to link molecular building blocks together or to introduce functional groups. In this section, different types of concurrent polymerization methods, as well as concurrent side group modification during ATRP, are discussed.

### 2.1. Concurrent ATRP and ROP

**Figure 2:**
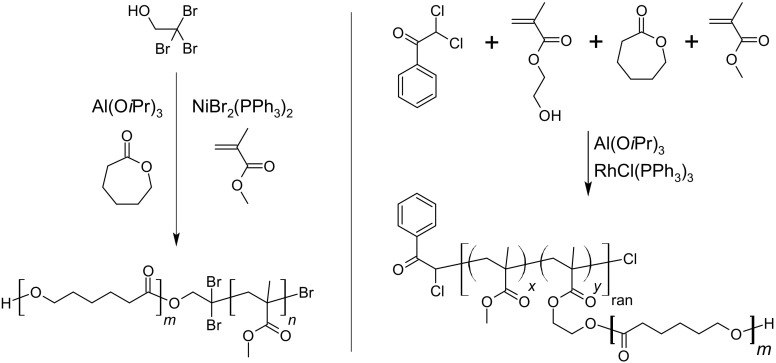
Synthesis of (**left**) diblock and (**right**) graft copolymer by concurrent ATRP and ROP [44].

In Ru, Fe, and Ni catalyzed ATRP [43], metal alkoxides, such as Al(O*i*Pr)_3_, are commonly used to improve the reaction activity and the control over the polymerization. Since the metal alkoxide, which is normally a Lewis acid, could also be used as the catalyst for ROP of ϵ-caprolactone (CL), it was found that ROP and ATRP could be carried out in one pot to produce both types of polymers simultaneously. When tribromoethyl alcohol was used as the initiator, in the presence of NiBr_2_(PPh_3_)_2_ and Al(O*i*Pr)_3_ as the dual catalysts, poly(ϵ-caprolactone) (PCL) and poly(methyl methacrylate) (PMMA) were synthesized concurrently to form a diblock copolymer, Figure 2 (left) [44,45]. By adjusting the reaction temperature, i.e., 60 ${}^{{}^{\circ}}C$ to 75 ${}^{{}^{\circ}}C$, and the reaction time, i.e., 1 h to 20 h, block copolymers with different fractions of CL units, i.e., 48% to 85%, and different molecular weights, i.e., 11,000 to 26,000, were obtained. The molecular weight distributions were relatively broad resulting in $M_{w}/M_{n}$ values from 1.3 to 2.3. Instead, if 2-hydroxyethyl methacrylate (HEMA), CL, and Al(O*i*Pr)_3_ were added to a regular ATRP system, graft copolymers could be synthesized in a one-step approach. Since HEMA played the role as both the ATRP monomer and the ROP initiator, it was incorporated into the main chain of the graft polymer via ATRP and initiates ROP of CL to form the side chains, Figure 2 (right) [44]. Similarly, Cu catalyzed concurrent ATRP of styrene (St) and ROP of CL in the presence of Sn(Oct)_2_ was reported to synthesize the diblock copolymer in one-pot [46]. More recently, it was found concurrent ATRP/ROP could be performed in the absence of metal catalysts. Environmentally friendly organic catalysts, i.e., perylene and P_2_−*t*−Bu, were used in this report and resulted in successful concurrent polymerization under sunlight radiation giving PMMA-*b*-PCL diblock copolymers in one-pot using a dual initiator [47].

**Figure 3:**
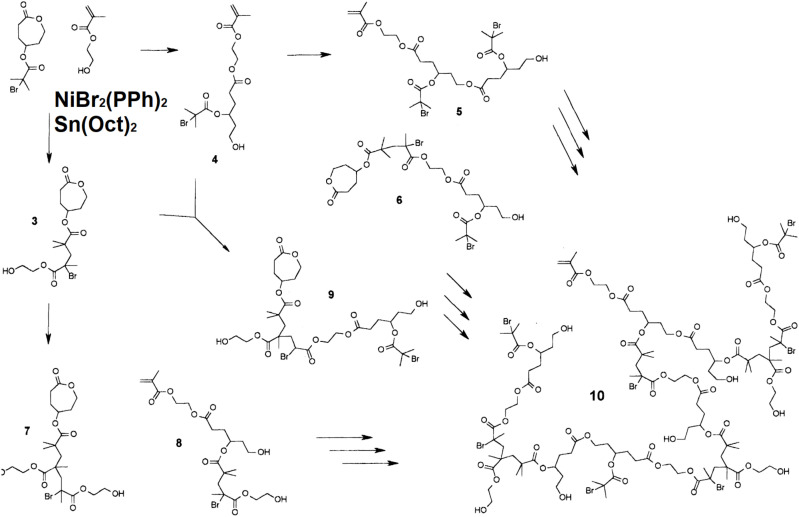
Concurrent atom transfer radical polymerization (ATRP) and ring-opening polymerization (ROP) for branched copolymer synthesis. Adapted with permission from [48], Copyright© 1999 American Chemical Society.

More interestingly, if the concurrent polymerization was carried out with an ROP monomer bearing an ATRP initiation site, i.e., (γ-(ϵ-caprolactone) 2-bromo-2-dimethylpropionate), and an ATRP monomer bearing an ROP initiation site, i.e., HEMA, branched copolymers with two types of backbones, i.e., polyester and polymethacrylate, could be obtained. The branching architecture could be easily adjusted by varying the monomer ratio, and the addition of the appropriate ROP monomer, i.e., CL, and ATRP monomer, i.e., methyl methacrylate (MMA), Figure 3 [48].

### 2.2. Concurrent ATRP and ROMP

**Figure 4:**
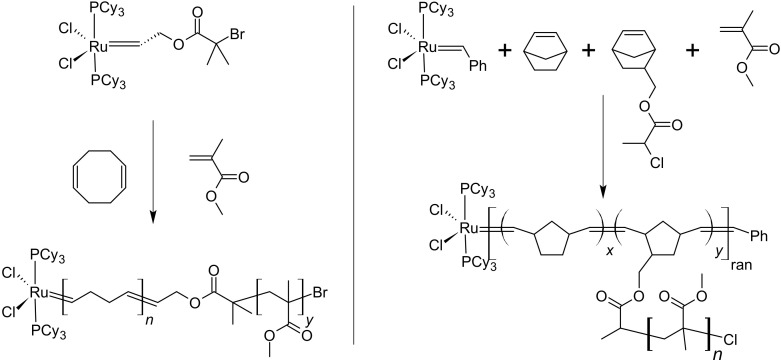
Synthesis of (**left**) diblock and (**right**) graft copolymer by concurrent ATRP and ring-opening metathesis polymerization (ROMP) [49,50].

Ru catalyst has been used widely in both ROMP and ATRP. It was interesting that the same Ru catalyst could catalyze both types of polymerizations. More importantly, when an alkylidene type Ru catalyst was covalently linked with an ATRP initiator, it could catalyze ROMP and ATRP concurrently and produce diblock copolymers in one-step reaction, Figure 4 (left) [49]. At 65 ${}^{{}^{\circ}}C$, with the same initial concentrations of both monomers, i.e., 1,5-cyclooctandiene (COD) and MMA, ROMP of COD, and ATRP of MMA had very similar polymerization rates in the presence of excess tricyclohexylphosphine (PCy_3_). By adjusting the ratio of monomers and the reaction time, diblock copolymers with number average molecular weights from 7700 to 17,300 and fractions of MMA ranging from 38% to 77% were obtained. The molecular weight distribution was similar, i.e., $M_{w}/M_{n}=1.5$ to 1.6 in all cases. Alternatively, if the ROMP monomer was covalently linked with an ATRP initiator, the concurrent polymerization would result in graft copolymers [50], or star-like copolymer [51]. It was reported microemulsion concurrent ATRP/ROMP was carried out with the first generation Grubbs catalyst, Figure 4 (right) [50]. A functional ROMP monomer, i.e., norbornene (NB) covalently linked with an ATRP initiation site, was used in this report. NB and MMA were used as the monomers. As a result, the polymers had PNB backbones and PMMA as the side chains. The microemulsion polymerization method resulted in relatively high molecular weights, i.e., $M_{n}$ = 50,000 to 100,000, with $M_{w}/M_{n}$ around 2. It is worth mentioning that in the concurrent polymerization the monomer unit fraction in the polymer product was very close to the monomer fraction in the reactant mixture when the ratio of NB changed from 8% to 47%.

### 2.3. Concurrent ATRP and RAFT

**Figure 5:**
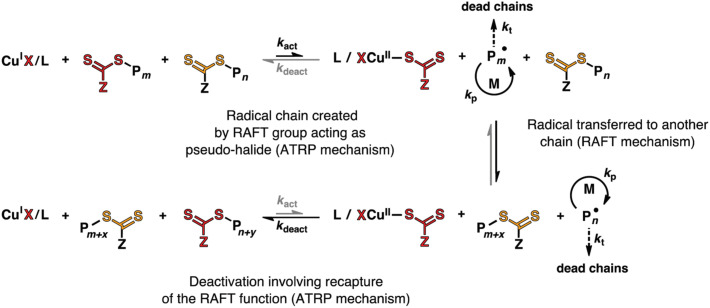
Concurrent ATRP and reversible addition-fragmentation chain-transfer polymerization (RAFT) mechanism. Adapted with permission from [20], Copyright© 2017 Royal Society of Chemistry.

Though alkyl halide initiators are most widely used in ATRP, compounds with pseudo halogen groups, such as dithiocarbamate and dithioester, can also generate radicals in the presence of transition metal catalysts by the transfer of the pseudo halogen group and the oxidation of the transition metal to a higher oxidation state. Dithiocarbamate (DC) type of compounds was used as the initiators for ATRP of styrene (St) and MMA. The control over the polymerization was excellent. In both cases, the number average molecular weights of the polymers increased linearly with the monomer conversion and relatively narrow molecular weight distribution, i.e., $M_{w}/M_{n}<1.2$, was achieved [52]. In these processes, the reversible deactivation by DC group transfer should have dominated the molecular weight regulation among polymer chains. However, reversible chain transfer should also occur since DC groups are known as moderate chain transfer agents. Thus, the reaction mechanism was more likely a combination of ATRP and RAFT with higher ATRP component [53]. When Cu(I) catalyst was used with efficient RAFT agents, both ATRP and RAFT components were important in the reaction mechanism, Figure 5 [54]. In the concurrent ATRP/RAFT process, the Cu(I) catalysts with N,N,N′,N″,N″-Pentamethyldiethylenetriamine (PMDETA), tris[2-(dimethylamino)ethyl]amine (Me_6_TREN), or 2,2′-bipyridine (bpy) as the ligands induced the radical generation via reversible group transfer, while the reversible chain transfer to the dithioester chain end was employed to regulate the molecular weight distribution. The polymerization was controlled by two concurrent reaction mechanisms. Similar to SARA ATRP [27], and *e*ATRP [30], the concurrent polymerization could be carried out with trace amount of Cu catalyst, as low as 10 ppm, by regenerating Cu(I) with metallic Cu or electricity [55,56]. In addition to Cu, Fe catalysts could also be used in concurrent ATRP/RAFT [57]. Another interesting application of the synergy between ATRP and RAFT is the in situ formation of RAFT agent and concurrent polymerization [58,59]. In the presence of copper catalyst, alkyl halide, and bis(thiocarbonyl) disulfide, the RAFT agent could be synthesized with very high efficiency and purity. Thus, the in situ generated RAFT agent could be used directly for the polymerization and result in well-controlled molecular weights and narrow molecular weight distributions.

**Figure 6:**
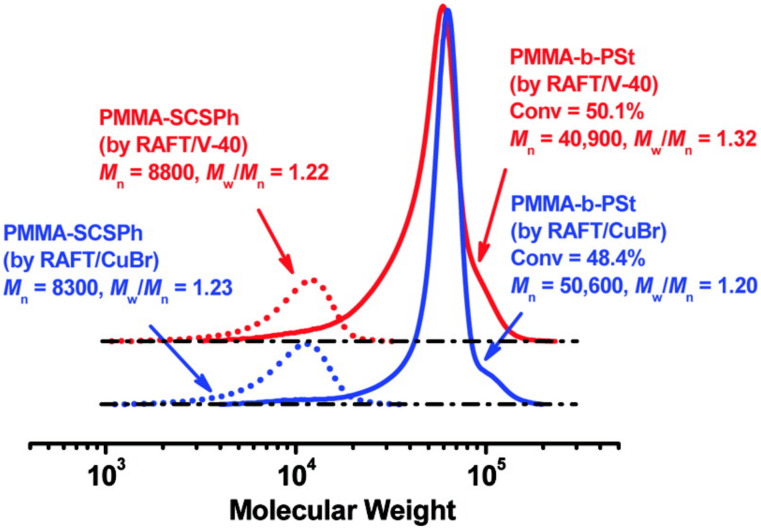
Concurrent ATRP and RAFT to eliminate new chain generation. Adapted with permission from [54], Copyright© 2008 American Chemical Society.

Normally, a RAFT polymerization requires a thermal initiator to generate radicals and to sustain the polymerization, which means new polymer chains are produced constantly. In the case of diblock copolymer synthesis, the introduction of new chains also means a small fraction of homopolymers would be produced. By using concurrent ATRP/RAFT, the radicals are produced by group transfer rather than from thermal initiators, thus the introduction of new polymer chains could be avoided. For comparison, two samples of PMMA macroinitiators were synthesized under typical RAFT conditions with 1,1′-azobis(cyclohexanecarbonitrile) (V-40) and concurrent ATRP/RAFT with Cu(I), respectively. These two polymerizations were carried out at the same temperature for the same reaction time and to achieve very similar monomer conversions. Then both macroinitiators were chain extended with St under either RAFT/V-40 or ATRP/RAFT conditions. In the case of RAFT/V-40 condition, the block copolymer PMMA-*b*-PSt had a relatively high $M_{w}/M_{n}$ value of 1.3 and the gel permeation chromatography (GPC) trace showed a tailing towards low molecular weight indicating the formation of low molecular weight dead chains. On the other side, the PMMA-*b*-PSt block copolymer synthesized under ATRP/RAFT condition showed a narrower molecular weight distribution, i.e., $M_{w}/M_{n}=1.2$, as well as a more symmetric GPC curve. The chain end functionality analysis revealed the ATRP/RAFT condition helped to maintain a higher level of living chains giving a high chain-end functionality ∼93% compared to that of ∼75% under RAFT/V-40 conditions. The above results indicate that a purer block copolymer, i.e., less small molecular weight homopolymers, was prepared using the concurrent ATRP/RAFT system than the conventional RAFT system, Figure 6 [54]. As a result that the formation of new chains from radical initiators is suppressed, as compared to typical RAFT polymerization, concurrent ATRP/RAFT in the presence of Cu(0) could be employed to synthesize ultrahigh molecular weight PMMA and polymethacrylate block copolymers with relatively narrow molecular weight distribution, i.e., $M_{n}>10^{6}$ and $M_{w}/M_{n}=1.2$ to 1.3 [55].

### 2.4. Concurrent ATRP and ATRA Step-Growth Polymerization

In ATRP, the activation of the alkyl halide, the monomer addition, and the deactivation take place repeatedly. However, if the new generation of alkyl halide becomes very inactive after the addition of the monomer, i.e., it could not be activated again by the transition metal, the addition would happen only once. This type of reaction is called atom transfer radical addition (ATRA), which is a very useful reaction in organic chemistry [41]. By using a monomer bearing an active alkyl halide group and an unconjugated C=C group, a new type of polymerization was carried out. Similar as in ATRP, the alkyl halide was activated to generate a radical, the vinyl group could react with the radical by addition. However, after deactivation of the radical by halogen transfer, the newly formed alkyl halide became inactive and could not participate in further reactions. Thus, the monomers were linked together in a stepwise addition by ATRA rather than chain-growth polymerization [60].

**Figure 7:**
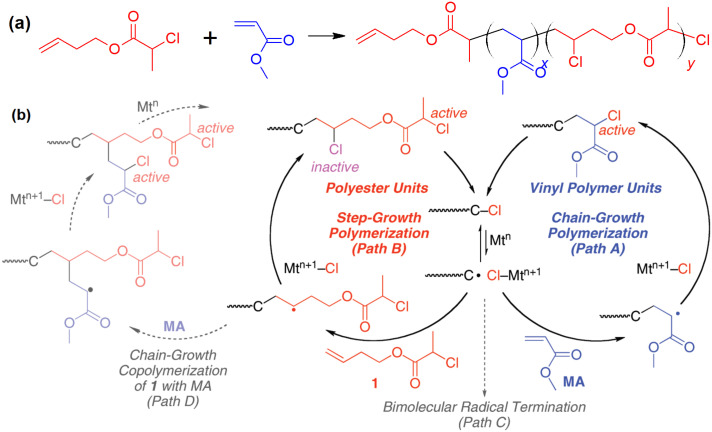
Concurrent ATRP and atom transfer radical addition (ATRA) step-growth polymerization. (**a**) The monomers and the structure of the polymer; (**b**) reaction mechanisms involved in the polymerization. Adapted with permission from [61], Copyright© 2010 American Chemical Society.

When a regular ATRP monomer, e.g., an acrylate, was added to the reaction system mentioned above, a concurrent step-growth and chain-growth polymerization took place, Figure 7 [61]. Since the addition of acrylate was much faster than the addition of the unconjugated vinyl group, each time of the activation of the alkyl halide resulted in the addition of a number of acrylate monomers and then one or a few unconjugated vinyl group. Then the radical would be deactivated and form an inactive alkyl halide. Thus, the polymer formed in this concurrent polymerization should consist of sections of polyacrylate separated by ester units in the main chain. By adjusting the ratio of two types of monomers, the sequence length of the polyacrylate could be varied.

**Figure 8:**
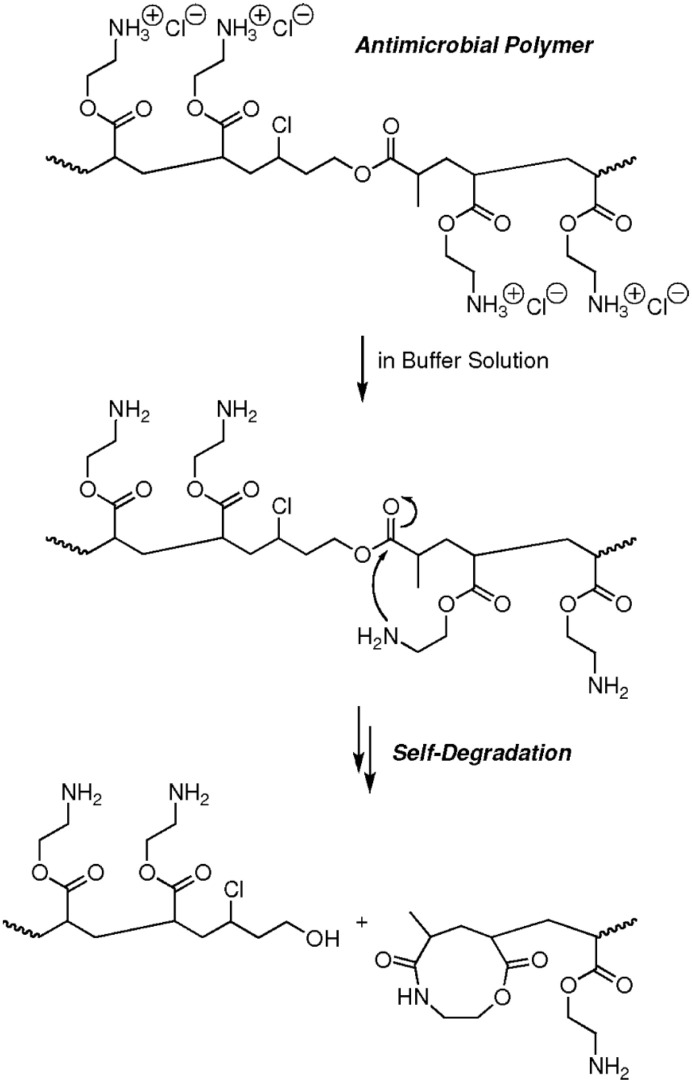
Polymers with degradable units synthesized by concurrent ATRP and ATRA step-growth polymerization. Adapted with permission from [63], Copyright© 2012 American Chemical Society.

This kind of polymerization is very useful for introducing functional motifs into the main chain of the polymers. For example, the simultaneous polymerization of N-isopropylacrylamide (NIPAM) and an ester-linked monomer was carried out in aqueous media at ambient temperature. The obtained copolymers could be easily degraded by the cleavage of the ester linkage in the main chain. The degradability and thermoresponsibility of the polymer could be tuned by changing the monomer structure or initial feed ratios [62]. Further, self-degradable antimicrobial copolymers bearing cationic side chains and main chain ester linkages were synthesized using the concurrent ATRP and ATRA step-growth polymerization. The polyacrylate type copolymer with primary amine side chains could degrade to lower molecular weight oligomers due to the amidation of the ester groups in the main chain by the nucleophilic addition of primary amine groups in the side chains resulting in cleavage of the polymer main chain, Figure 8 [63].

### 2.5. Concurrent ATRP and CuAAC Click Chemistry

ATRP and CuAAC are often used together in macromolecular architecture design [19]. The reasons are (1) polymers synthesized by ATRP have halogen chain ends which can be converted to azide groups easily; (2) both alkyne and azide groups are stable during ATRP as long as they do not coexist in the same reaction; (3) both ATRP and CuAAC use Cu(I) as the catalyst. In reality, many ATRP catalysts can be employed directly for CuAAC. It is a natural question of whether ATRP and CuAAC could be conducted concurrently. The answer is positive. Actually, concurrent ATRP/CuAAC has been used as a very efficient tool to synthesize block copolymers [64], brush polymers [65,66,67], polymers with functional side groups [68,69], networks [70], etc.

**Figure 9:**
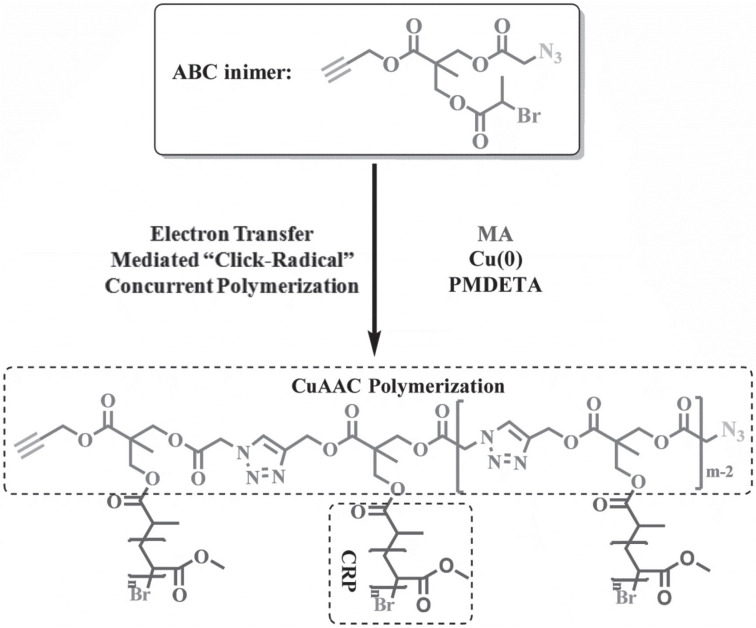
One-step synthesis of brush polymer from small molecules by concurrent ATRP and copper-catalyzed azide-alkyne cycloaddition (CuAAC) click polymerization. Adapted with permission from [65], Copyright© 2017 WILEY-VCH Verlag GmbH & Co. KGaA, Weinheim.

One-step synthesis of brush polymer directly from small molecules was successfully carried out using a tri-functional inimer in the presence of Cu(0)/pentamethyldiethylenetriamine (PMDETA) as the catalyst and methyl acrylate (MA) as the monomer, Figure 9 [65]. The inimer exhibited one alkyne group and one azide group, thus could polymerize via step-growth click polymerization [71]. In addition, the inimer also bore an ATRP initiation site and could initiate polymerization of MA. As a result, a brush polymer was obtained in one-step.

**Figure 10:**
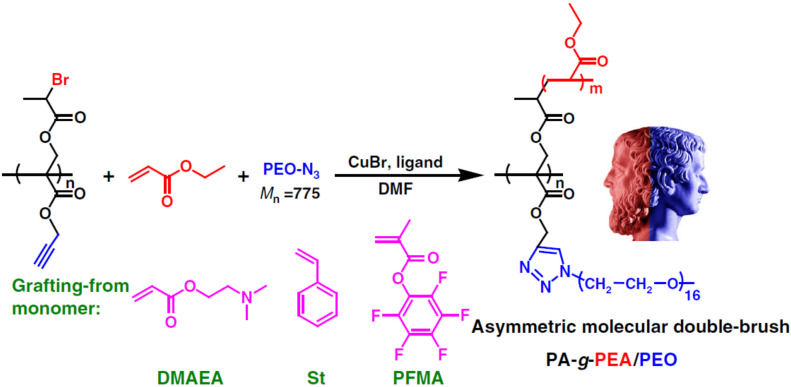
Synthesis of Janus armed bottlebrush copolymer by concurrent ATRP and CuAAC click chemistry. Adapted with permission from [67], Copyright© 2017 Springer Nature.

The simultaneous ATRP and CuAAC made the synthesis of complex macromolecular architectures significantly easier. For example, Janus armed bottlebrush copolymer could be synthesized conveniently by employing concurrent ATRP/CuAAC, Figure 10 [67]. The key of the success was the application of an acrylate type monomer bearing both an alkyne group and an ATRP initiation site, i.e., *tert*-butyl 2-((2-bromo-propanoyloxy)methyl)acrylate (*t*BBPMA). The monomer *t*BBPMA was polymerized by RAFT to get a polymer with two types of functional side groups on each monomer unit. Finally, the Janus armed bottlebrush copolymer was synthesized in one-pot by concurrent ATRP of vinyl monomers and CuAAC coupling of poly(ethylene oxide) with an azide end group (PEO−N_3_). Well-defined asymmetric bottlebrush copolymers with controlled architecture and narrow molecular weight distributions, i.e., $M_{n}∼$100,000 and $M_{w}/M_{n}\leq 1.31$, were obtained.

**Figure 11:**
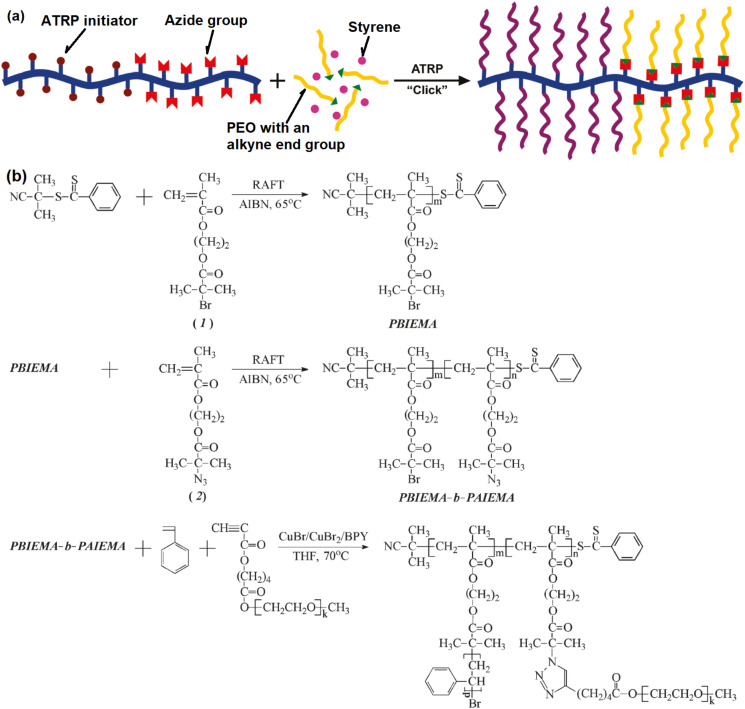
(**a**) Schematic illustration, and (**b**) reaction mechanism of the synthesis of bottlebrush block copolymer by concurrent ATRP and CuAAC click chemistry. Adapted with permission from [66], Copyright© 2011 American Chemical Society.

Similarly, bottlebrush block copolymers could be synthesized by concurrent ATRP/CuAAC [66], Figure 11. Usually, the synthesis of bottlebrush block copolymers involves the synthesis of a diblock copolymer with different functional side groups on each block, and the polymerization of two types of side chains individually via two kinds of polymerization mechanisms [72]. The application of concurrent ATRP/CuAAC could make the procedure easier. Firstly, a linear diblock copolymer was synthesized by RAFT with one block bearing ATRP initiation sites as the side groups and the other block bearing azide groups. Then, the two types of side chains were grafted from and onto the backbone simultaneously to get the bottlebrush block copolymer in one-pot via ATRP of St and CuAAC of poly(ethylene oxide) with an alkyne end group (PEO−#). Well defined bottlebrush copolymer was obtained with $M_{n}∼170,000$ and relatively narrow molecular weight distribution, i.e., $M_{w}/M_{n}=1.13$.

**Figure 12:**
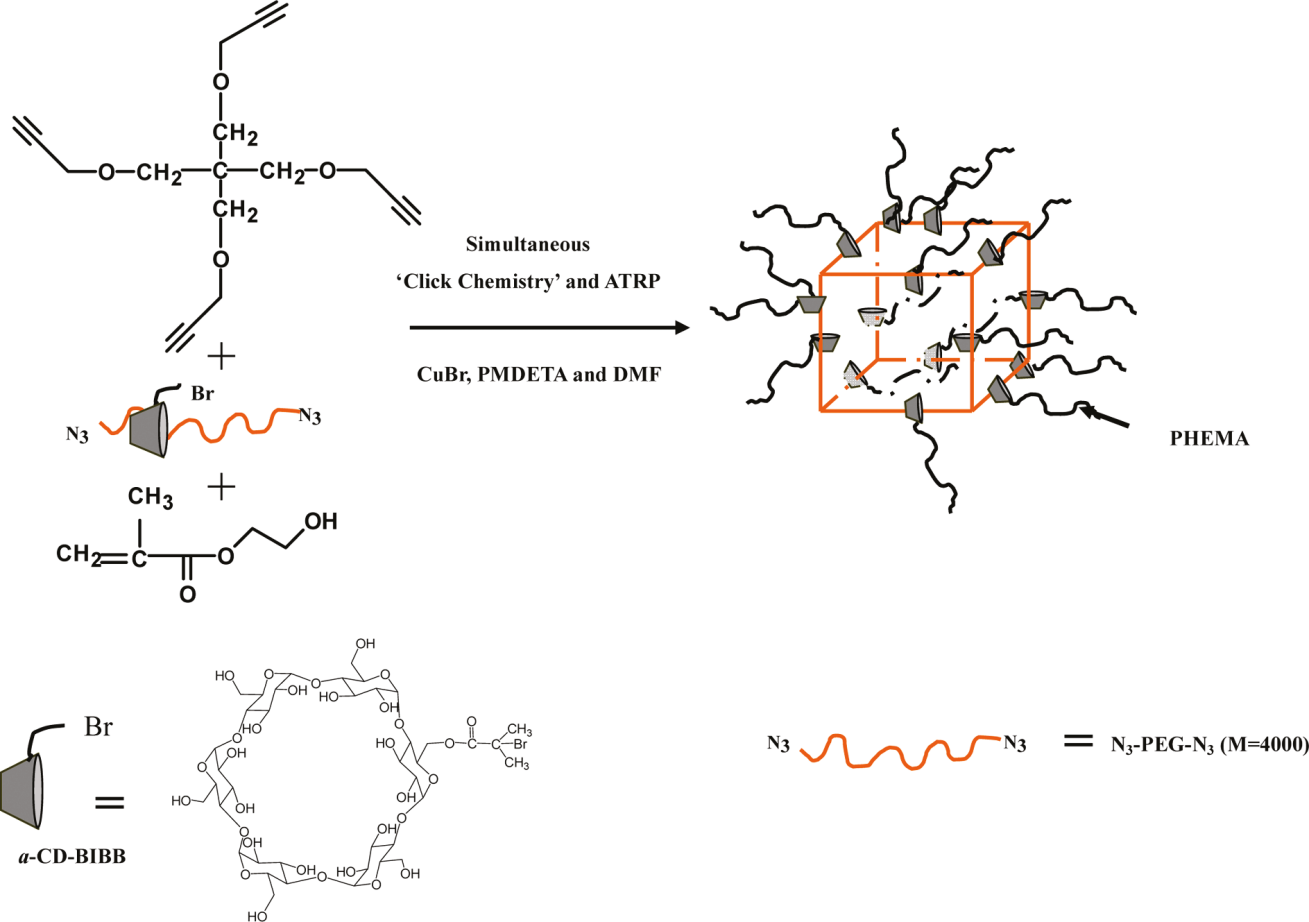
Sliding-graft interpenetrating polymer network synthesized by concurrent ATRP and CuAAC click chemistry. Adapted with permission from [70], Copyright© 2010 American Chemical Society.

Another interesting example showed the synthesis of a semi-interpenetrating polymer network (s-IPN) by concurrent ATRP/CuAAC. Firstly, α-cyclodextrin (α-CD) with an ATRP initiator was combined with poly(ethylene glycol) having two azide chain end functional groups (N_3_−PEG−N_3_). PEG and α-CD could form supramolecular structures with the PEG polymer chain inserted into the rings of α-CD. Then the click polymerization of N_3_−PEG−N_3_ with a tetra-functional monomer bearing four alkyne groups took place simultaneously with ATRP of 2-hydroxyethyl methacrylate (PHEMA). As a result, movable sliding-grafted poly(2-hydroxyethyl methacrylate) (PHEMA) on the crosslinked network was formed as s-IPN-PEG/R-CD-sg-PHEMAs, Figure 12 [70]. The s-IPN-PEG/R-CD-sg-PHEMAs had a well-defined PEG network with uniform and tunable sliding-grafted PHEMA chains. Most importantly, the diffusion of interpenetrated PHEMA from the network was prevented because the PHEMA brushes were topologically fixed on the PEG networks.

### 2.6. Concurrent ATRP and Side Group Modification

**Figure 13:**
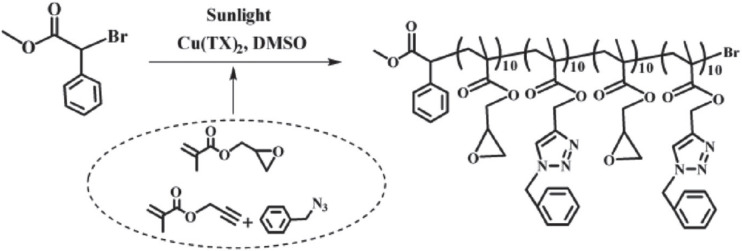
Concurrent ATRP and side group modification by CuAAC click chemisty. Adapted with permission from [68], Copyright© 2017 WILEY-VCH Verlag GmbH & Co. KGaA, Weinheim.

CuAAC can be used as a tool for side group modification by combining small functional molecules onto the polymers. The polymerization and side group modification could be completed simultaneously. One example showed ATRP of a monomer with an alkyne group could couple with a functional molecule with an azide group concurrently during the polymerization, Figure 13 [68]. More importantly, the reaction could be performed under open-to-air conditions via photo (sunlight)-induced electron transfer ATRP, utilizing one-component Cu(II) thioxanthone carboxylate as multifunctional photocatalyst and oxygen scavenger.

**Figure 14:**
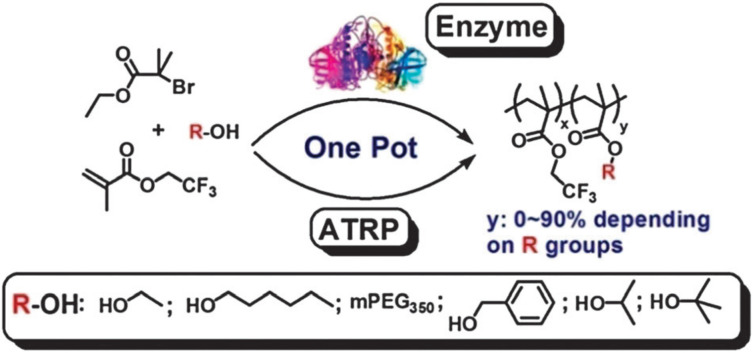
Concurrent ATRP and side group modification by enzyme catalyzed transesterification. Adapted with permission from [73], Copyright© 2012 Royal Society of Chemistry.

Enzyme catalyzed transesterification has also been used for concurrent side group modification during ATRP. The activity of the enzyme could be maintained under the polymerization conditions [73]. When primary alcohols were used, the enzyme cooperated well with ATRP, and maintained nearly complete activity after polymerization. By using different alcohols as substrates, the in situ monomer transformation resulted in copolymers possessing different transformed –R side groups. In addition, optically active polymers were synthesized using this method since the enzyme-catalyzed transesterification had regioselectivity, Figure 14 [74]. A chiral monomer with >99% ee value was achieved proving the enzymatic resolution efficiency of racemic alcohol under polymerization conditions was excellent. The optically active polymers obtained from the concurrent polymerization and transesterification were enantiomerically pure. The optical properties of the polymers synthesized by the concurrent method were the same as those of the homopolymer from direct polymerization of the chiral monomer.

**Figure 15:**
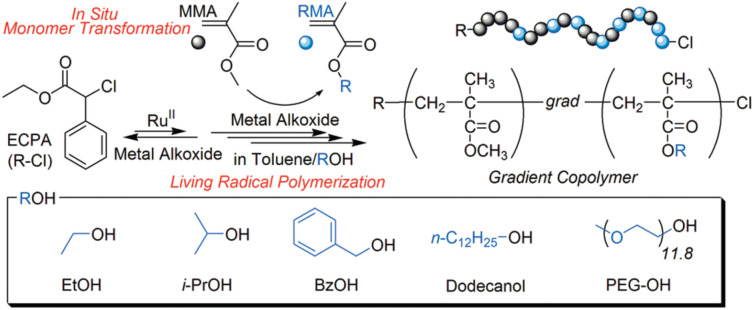
Synthesis of gradient copolymer by concurrent ATRP and selective transesterification. Adapted with permission from [75], Copyright© 2009 American Chemical Society.

Another example showed the in situ transesterification of MMA to other functional methacrylate monomers with metal alkoxide as the catalyst. Interestingly, once MMA was polymerized, the monomer unit became inactive and the transesterification would not occur. Thus, the concurrent reaction provided a smart way to synthesize gradient copolymer, because the ratio of MMA in the monomer mixture decreased continuously, Figure 15 [75]. This methodology could be further expanded to synthesize block, random, random-gradient, and gradient-block copolymers by the sequence regulation of multi-monomer units [76].

## 3. Future Research Directions

Simplifying the reaction procedure is obviously a significant advantage of concurrent polymerization. Several representative examples have been discussed in the previous sections, including the synthesis of block copolymers, brush polymers, networks, and polymers with functional side groups. Other advantages of concurrent polymerization are worth mentioning as well. In some cases, concurrent polymerization can help to improve the quality of the product as shown in the case of concurrent ATRP/RAFT, Figure 6. In some other cases, special molecular architectures or synthetic strategies could be easily achieved by using concurrent polymerization, for example, the synthesis of branched polymers with two types of backbones, Figure 3; the incorporation of ester units in ther polyacrylate, Figure 7; the in situ selective transesterification of monomers, Figure 15, etc. It is very attractive to use concurrent polymerization methods whenever they are applicable. However, the application of concurrent polymerizations also has some limitations. First of all, the two reactions happen simultaneously must be either orthogonal, e.g., in the case of ATRP/CuAAC, or interactive in a constructive way, e.g., in the case of concurrent ATRP/RAFT. Secondly, the two reactions are performed under the same temperature, pressure, and solvent, and they are expected to have comparable reaction rates, thus could produce the targeted product within a similar time frame. To fulfill all the requirements is not always a trivial task. Optimization of the reaction conditions is critical and could be very challenging compared to optimizing the reaction conditions for a single reaction. Yet, many successful concurrent polymerization methods have been developed as shown above. It is expected that in the future (1) more concurrent polymerization methods will be developed; (2) optimized reaction conditions will be addressed to achieve high-quality products with well-defined and precisely tailored molecular structures; (3) more applications of concurrent polymerizations in advanced functional material development will be witnessed.

## 4. Summary

In summary, ATRP combining with other concurrent reactions has been employed as a powerful tool in macromolecular engineering. The concurrent polymerization methods not only can reduce the complexity of the synthetic procedure, but also can lead to higher quality products or special molecular architectures or synthetic strategies. It is challenging but also very attractive to optimize available concurrent polymerization methods or to develop new ones. More successful concurrent polymerization methods are expected in the future to enrich the toolbox for efficient macromolecular engineering. On one hand, by applying the highly efficient synthetic strategies, some complex macromolecular structures with special properties could be explored further since the difficulty of the synthesis could be significantly reduced. On the other hand, some novel molecular structures which are currently not accessible will be developed resulting in entirely new functional materials.
